# The quality of voluntary medical male circumcision done by mid-level workers in Tshwane District, South Africa: A retrospective analysis

**DOI:** 10.1371/journal.pone.0190795

**Published:** 2018-01-19

**Authors:** Sanele Ngcobo, Jacqueline Elizabeth Wolvaardt, Martin Bac, Elize Webb

**Affiliations:** 1 School of Health Systems and Public Health, Faculty of Health Sciences, University of Pretoria, Pretoria, South Africa; 2 Department of Family Medicine, School of Medicine, Faculty of Health Sciences, University of Pretoria, Pretoria, South Africa; Boston University School of Public Health, UNITED STATES

## Abstract

**Background:**

Voluntary medical male circumcision (VMMC) reduces the acquisition of human immunodeficiency virus (HIV) in heterosexual men by up to 60%. One HIV infection is averted for every 5 to 15 VMMCs. To conduct VMMCs in large populations, large numbers of trained healthcare professionals are needed. Countries in Sub-Saharan Africa have a high burden of HIV and a shortage of healthcare professionals, creating a healthcare conundrum. To bridge this gap, South Africa launched a new cadre of mid-level medical worker called Clinical Associates (CA). We assessed the ability of CAs to perform circumcisions of adequate quality and their subsequent usefulness to meet the demands of VMMCs in a population with a high HIV burden.

**Methods:**

We conducted a retrospective analysis, reviewing patient files (n = 4850) of surgical VMMCs conducted over a 16-month period. Patient files were sourced from clinics and hospitals that provided free VMMCs in Tshwane district in South Africa.

**Findings:**

Clinical associates performed 88.66% of the circumcisions and doctors performed the remaining 11.34% (p < 0.001). The number of adverse events did not differ between the two groups. Data on intra-operative adverse events were available for 4 738 patients. Of these, 341 (7.2%) experienced intra-operative adverse events. For the whole sample, 44 (8.1%, n = 543) adverse events occurred during circumcisions done by doctors and 297 (7.1%, n = 4195) occurred during circumcisions done by CAs (p = 0.385). Clinical associates performed circumcisions in shorter times (duration: 14.63 minutes) compared to doctors (duration: 15.25 minutes, t = -7.46; p < 0.001). Recorded pain, bleeding, swelling, infection and wound destruction did not differ between clients circumcised by CAs and doctors. This study is limited by the use of data from a single district.

**Conclusions:**

Clinical associates contribute to the demands for high numbers of VMMCs in Tshwane district, South Africa. Clinical associates perform VMMCs at a clinical standard that is comparable to circumcisions performed by doctors.

## Introduction

Voluntary medical male circumcision (VMMC) reduces the transmission of human immunodeficiency virus (HIV) in heterosexual men by up to 60%.[1–3] Ultimately averting 1 HIV infection for every five to 15 VMMCs performed.[4] The World Health Organization (WHO) and the Joint United Nations Programme on HIV/AIDS (UNAIDS) recommend that VMMC be offered to heterosexual men in combination with other effective HIV risk reduction interventions in settings with generalized HIV epidemics such as South Africa (SA).[4] South Africa, having a high burden of HIV and the highest rates of new transmissions, is a priority country to scale up the rate of VMMCs and aims to achieve the WHO target of a circumcision coverage of 80%.[5] In 2011, SA needed 4,3 million more circumcisions among men aged 15 to 49 years to reach the recommended coverage by 2016.[5]

One of the challenges of scaling up VMMCs is a shortage of human resources. Sufficient numbers of available healthcare professionals are needed to ensure surgical and non-surgical efficiency gains of low cost procedures such as VMMCs.[5] In 2008, the South African National Department of Health (NDoH) launched a new cadre of mid-level medical worker called Clinical Associates (CAs) to increase the number of healthcare professionals in the public sector, particularly in rural areas.[6] Clinical associates are similar to physician assistants in other parts of the world. With more than 516 graduates deployed in eight provinces in 2015, CAs could be critical in the provision of VMMCs. To address the short supply of medical doctors in the public sector, the NDoH has identified task shifting of VMMCs from medical doctors to CAs to meet the demands of this prevention strategy.[7] Task shifting is the rational redistribution of tasks[8] from a more qualified health worker to a less qualified health worker with the skills and knowledge to perform that particular task.[9] Task shifting of VMMC has been successful in other African countries[10–12] and has reduced costs.[13,14] In particular, CAs should contribute to the health workforce by diagnosing and managing common conditions and minor surgical procedures such as VMMCs.

### VMMC training

The Centre for HIV and AIDS Prevention Studies (CHAPS), in collaboration with the Foundation for Professional Development (FPD), provides quality assurance and training for HIV-related programs and VMMC in Africa.[15] Doctors and CAs interested in performing VMMC are trained to conduct VMMCs and professional nurses are trained to assist both CAs and doctors.[15] The training covers both theoretical and practical components of VMMC, including pre-operative assessment (history taking, physical examination and specifically genital examination), procedure for preparation of the VMMC kit, the surgical procedure, post-operative care and follow up care of any adverse events.

### VMMC adverse events

Reported adverse event rates in VMMC trial studies are 3.8% in SA, 3.6% in Uganda and 1.5% in Kenya.[16] These adverse event rates are favorable compared to adverse event rates in traditional circumcisions, which are as high as 35.2%.[17] While the adverse event rates associated with VMMC in developing countries are low compared to traditional circumcisions, they are higher than the VMMC adverse event rates reported in developed countries (0.2% to 0.6%). Adverse events are prevented by pre-screening clients and using a sterile surgical technique. [18] Classification and treatment for adverse events associated with VMMC are outlined in an Adverse Event Action Guide. [19]. Excessive bleeding (intra- and post-operative), infection, wound disruption and pain (intra- and post-operative) are common adverse events. [19]

The quality and clinical outcomes of VMMCs can be measured by measuring the presence and severity of these common adverse events. The NDoH in SA has scaled up the provision of VMMCs through task shifting from medical doctors to CAs to meet the high target for VMMC coverage. We conducted a retrospective analysis, measuring and comparing the presence of adverse events associated with VMMCs conducted by CAs and doctors. This provides a measure of quality and sustainability for enhanced provision of VMMCs using a task shifting approach.

## Methods

We conducted a retrospective analysis among the Tshwane District’s clinics and hospitals that provided free VMMCs during the 16-month study period. Tshwane District has a large population and many VMMC sites that provide free services. All the centres in this study are supported by CHAPS. We analyzed records from the January 2014 onwards to encapsulate the impact of CAs on the provisioning of VMMCs. Prior to this, very few CAs had entered the open job market.

### Sample size

We sampled all 4 850 files of clients who underwent surgical VMMCs. Data from client files were divided into 2 groups, those circumcised by doctors and those circumcised by CAs. Files were included if clients had a VMMC in the 16 months of the study period and were 10 years or older at the time of circumcision. Files with no client name or type of healthcare professional were excluded.

### Data collection

We designed a data collection tool based on the information recorded in the standardized CHAPS’ client files. Using the data collection tool, we recorded information from the pre-operative assessment, the intra-operative procedure and post-operative information including any adverse events. For each category of healthcare professional, we recorded the number of procedures and their durations. We recorded patient age, pre-existing conditions such as diabetes and pre-existing penile conditions such as phimosis. We recorded any intra- or postoperative adverse events such as swelling and bleeding. All data were entered into an Excel 93(2003, Microsoft, One Microsoft Way, Redmond, Washington) spreadsheet by three trained data capturers. All the data capturers were computer literate and were trained on how to extract data from the clients’ files. The training included issues of confidentiality, data entry, review, storage, the data set code book and basic terms commonly used in the files. This training was led by the principal investigator. Each data capturer had to record at least 20 files under supervision to demonstrate competence. The principal investigator reviewed a random sample of patient files to ensure correct data extraction.

### Data analysis

The data were cleaned by the principal researcher with the assistance of a statistician. Data were analysed in STATA (version 14, 2015). Descriptive statistics are reported as means and standard deviations for parametric data. Categorical data were analysed using Chi square tests and numerical data using t-tests.

### Ethical considerations

The ethical approval certificate was granted by the University of Pretoria, Faculty of Health Science Research Ethics Committee (Ethics reference number: 421/2015). Permission to collect data was obtained from CHAPS. Each client had signed permission for the use of the data for research purposes. Client’s names were not captured during the data extraction process.

Confidentiality agreement forms were signed by the three data capturers. During fieldwork, the data were stored on password-protected laptops.

## Results

Clinical associates performed 88.7% (n = 4 300) of the circumcisions and the remaining 11.3% (n = 549) were done by doctors. The mean procedure duration for CAs was 15.27 min (SD ± 5.32) and 17.18 min for doctors (SD ± 7.38). Duration for CAs was significantly shorter than the duration of circumcisions performed by doctors (t = -7,46; p < 0.001). Clients circumcised by CAs were 19.03 years (SD ± 9.85) and clients circumcised by doctors were 17.71 years (SD± 9.31) old. Clients circumcised by CAs were significantly older (t = 2.98; p = 0.0029) than those circumcised by doctors (Table 1).

**Table 1:** The number of VMMCs performed, duration and study participants’ age.

	CLINICAL ASSOCIATES	DOCTORS	TOTAL	P VALUE
**Number of VMMCs done**	4,300 (88.7%)	549 (11, 3%)	4,849	< 0.001
**Mean duration of procedure**	15.27 min (SD = 5.32)	17.18 min (SD = 7.38)	15.49 min (SD = 5.62)	< 0.001
**Mean client age**	19.03 years (SD = 9.85)	17.71 years (SD = 9.31)	18.88 years (SD = 9.80)	< 0.001

We recorded pre-operative conditions, including phimosis (n = 58, 1.2%) and paraphimosis (n = 18, 0.4%). Doctors performed proportionately more circumcisions on clients with phimosis (X^2^ = 15.55; p<0.0001 and paraphimosis (X^2^ = 35.34; p<00001) compared to CAs. Doctors and CAs circumcised similar proportions of clients with other pre-existing conditions such as diabetes (X^2^ = 0.05; p = 0.815), hypertension (X^2^ = 0.007; p = 0.935) or HIV (X^2^ = 0.35; p = 0.554) (Table 2).

**Table 2:** Comparison of clients with pre-operative conditions circumcised by doctors and clinical associates (p<0.05 is significant).

CONDITIONS	CLINICAL ASSOCIATES N (%)	DOCTORS N (%)	TOTAL N (%)	CHI^2^	P VALUE
**Phimosis**	42 (1%)	16 (2.9%)	58 (1.2%)	15.55	0.000
**Paraphimosis**	8 (0.29%)	10 (1.8%)	18 (0.4%)	35.34	0.000
**Hiv +**	121 (3.7%)	12 (3.1%)	133 (3.6%)	0.35	0.554
**Diabetes**	10 (0.2%)	1 (0.2%)	11 (0.2%)	0.05	0.815
**Hypertension**	30 (0.7%)	4 (0.8%)	34 (0.7%)	0.007	0.935

Data on intra-operative adverse events were available for 4 738 circumcisions. Of these, 341 (7.2%) intra-operative adverse events were reported. From the whole sample, 8.1% (n = 44) occurred during the 543 circumcisions done by doctors and 7.1% (n = 297) during the 4 195 circumcisions done by CAs (X^2^ = 0.75; p = 0.385). Post-operative swelling within 2 days of the procedure was recorded for 1 460 clients, 30.2% (n = 1 299/4 195) were circumcised by CAs while 29.3% (n = 161/297) were circumcised by doctors. There were 53 (1.1%) cases of bleeding and 45 (1.1% of 4 195) were clients circumcised by CAs and 8 (1.5% of 297) were circumcisions done by doctors. There was no difference in adverse events such as swelling, pain, bleeding, infection and wound destruction between the circumcisions performed by CAs and doctors (Table 3).

**Table 3:** Comparison of the number of intra-operative and post-operative adverse events among clients circumcised by doctors and clinical associates (p<0.05 is significant).

	CLINICAL ASSOCIATES N (%)	DOCTORS N (%)	TOTAL N (%)	CHI^2^	P VALUE
**Any intra-operative adverse event**	297 (8.1%)	44 (7.1%)	341 (7.0%)	0.75	0.385
**Post-operative adverse events**					
**Swelling**	1 299 (30.2%)	161 (29.3%)	1 460 (30%)	0.19	0.666
**Pain**	69 (1.6%)	11 (2.0%)	80 (1.7%)	0.48	0.490
**Bleeding**	45 (1.1%)	8 (1.5%)	53 (1.1%)	0.76	0.384
**Infection**	20 (0.5%)	1 (0.2%)	21 (0.4%)	0.90	0.342
**Wound destruction**	13 (0.3%)	1 (0.2%)	14 (0.3%)	0.24	0.621

## Discussion

In the Tshwane District of South Africa, circumcisions performed by CAs were just as safe and effective compared with circumcisions performed by doctors. This finding is similar to the study done in Tanzania where the majority of the circumcisions was done by Clinical Officers with no difference in quality. [20] Clinical associates performed almost six times more circumcisions compared to doctors. Clients who were circumcised by CAs were significantly older than those circumcised by doctors. Clinical associates do not circumcise children younger than thirteen years old, as such the scope of practice may drive age differences in clients circumcised by doctors and CAs. [21] The clients’ general health did not differ between the two groups. Clinical associates spent less time doing circumcisions than doctors. Clinical associates may be more efficient and experienced due to high client volumes, alternatively doctors may be circumcising clients with complicated conditions such as phimosis and paraphimosis. Typically circumcision of clients with these conditions is more time-consuming.

The quality of care, as measured by the frequency of adverse events, and the procedure time was comparable between the two groups. No significant differences were found when a range of common intra-operative adverse events and post-operative adverse events were compared. The frequency of adverse events is comparable between CAs and doctors. Adverse events such as infection were similar to other studies.[12]

## Limitations

The use of routinely collected client information limited the examination of other possible measures of quality. The clinical record that was used at that time did not include the classification of any adverse events—only whether they occurred and when they occurred. This absence limited the researchers’ ability to draw any conclusions based on the severity of adverse events. Study results might not be generalizable to other populations as this study was only done in CHAPS-supported health facilities in one district. The correctness of the clinical information of patient files could not be validated.

## Conclusion

The current burden of HIV/AIDS in SA calls for more action in reducing the number of new infections and VMMC is one of those strategies. However there are not enough doctors in the public sector to circumcise the required numbers to achieve 80% coverage. We demonstrate that the clinical standard of VMMCs done by CAs is comparable to that provided by doctors, and that task shifting is justified. Clinical associates could play a pivotal role in the provision of VMMCs, especially in light of the many VMMCs that still need to be done.

## Supporting information

S1 Data (XLSX)
